# Chlorate of Potash as an Antiseptic and Germicide for the Mouth

**Published:** 1896-08

**Authors:** P. H. Unna


ARTICLE II.

### CHLORATE OF POTASH AS AN ANTISEPTIC AND GERMICIDE FOR THE MOUTH.

(Translated from *Monatshefte f. Praktische Dermasologie*, XVII. Band, 1893.)

In 1884 Professor Miller first published in the *Deutsche Medicintsche Wochenschrift* the results of his experiments as to the action of various antiseptic agents on bacteria cultures taken from the cavities of the mouth.

These tests interested me very much, mainly because my attention had previously been called to the constant activity of the schizomycetes in the formation of mercurial stomatitis. Having noticed the Miller's tables, which had formerly been generally referred to, made no mention of either chlorate or permanganate of potash, I suggested to the author the advisability of subjecting to an examination this class of oxydizing substances, particularly chlorate of potash, permanganate of potash and peroxide of hydrogen, in his future experiments. The idea that the bacteria of the mouth, accustomed to the continuous deoxydation taking place, might possibly be rendered less harmful by the employment of oxydizing agents, guided me.

Professor Miller was kind enough to entertain my proposition, and the following year published a number of new tables, all of which contained the three ingredients spoken of. It was noticed, however, that peroxide of hydrogen alone directly interfered with the growth of the fungi of the mouth; its destroying properties being second to that of corrosive sublimate and nitrate of silver. Permanganate of potash acted much weaker, and surprisingly weak was the action of chlorate of potash, mentioned at the end of the list of antiseptics. The latter is detrimental to the growth of schizomycetes only when used in a solution exceeding the proportion of 1 to 8.

The apparently unfavorable showing of Professor Miller's experiments is evidently responsible for the little importance at present attached to chlorate of potash in the manufacture of preparations for the preservation of the gums and the teeth, and I am confident that modern antisepsis in treatment of the mouth is fully in accordance with the principles set forth in the valuable and well known hand-book of Miller.

On page 226 Miller says:

"It is noteworthy that such useful agents as cholorate of potash and permanganate of potash possess such subordinate antiseptic properties; like iodoform, the favorable action of these remedies does not particularly depend upon their antiseptic value."

Notwithstanding the conclusion drawn from Miller's tables, I was still so fully convinced of the specific value of chlorate of potash in diseases of the mouth that I was reluctant to discard my favorite remedy. My own conclusion was that, if the efficiency of chlorate of potash as an antiseptic was to be enhanced, a much higher concentration would have to be used.

From that time on I discarded all solutions and employed pure chlorate of potash in the form of a tooth-paste containing 50 per cent. of the salt.

In this manner I achieved much better results than before, not only in mercurial stomatitis, but also in all diseases of the mouth, occasioned by the formation of schizomycetes.

During the past eight years in which I have employed chlorate of potash in this modified form I never had occasion to look for a better cleansing agent for the mouth, tonsils and teeth. Naturally, I fully agree with Miller that the efficacy of this remedy does not solely depend upon its antiseptic value.

It is my opinion that it possesses marked tonic properties acting favorably on mercurialized gums and imparting increased circulation. In many other affections of the mouth and tonsils the property of chlorate of potash of favoring secretions is particularly commendable.

To obtain this result, the pure chlorate of potash must be used; a small quantity is spread on the tooth-brush, applied to the teeth and gums and rubbed to a paste. After rinsing the mouth with clear water, a somewhat salty but refreshing taste remains.

I know of no other preparation that will remove so quickly and effectually the fetor oris, which is most apparent after meals and upon wakening in the morning. A number of cases in which this fetor proved sufficiently objectionable to enlist medical acid were promptly cured by the application of chlorate of potash after the patient had been treated for internal ailments.

Chlorate of potash, being a neutral salt, has absolutely no detrimental effect on the teeth; if used in the concentrated 50 per cent. form, it will promptly check the growth of the fungi for a long time, and in many instances destroy them entirely.

Chlorate of potash having been declared a poisonous chemical, it is safer to employ it in the form of tooth- paste; thus the possibility of an accident is excluded.

It has been demonstrated that the daily use of chlorate of potash tooth-paste is the very best prophylactic against caries of the teeth and affections of the tonsils, including diphtheria.—DR. P. H. UNNA, in *Notes and Remedies*.

